# Molecular characterization of a *Trichinella spiralis* elastase-1 and its potential as a diagnostic antigen for trichinellosis

**DOI:** 10.1186/s13071-020-3981-y

**Published:** 2020-02-24

**Authors:** Chen Xi Hu, Peng Jiang, Xin Yue, Jie Zeng, Xin Zhuo Zhang, Yan Yan Song, Ruo Dan Liu, Xi Zhang, Zhong Quan Wang, Jing Cui

**Affiliations:** Department of Parasitology, Medical College, Zhengzhou University, Zhengzhou, 450052 China

**Keywords:** *Trichinella spiralis*, Trichinellosis, Elastase-1, Serodiagnosis

## Abstract

**Background:**

*Trichinella spiralis* muscle larval (ML) excretion/secretion (ES) antigen is the most widely used diagnostic antigen of trichinellosis, but preparation of ES antigen requires collecting worms from infected animals, and detection of specific IgG against ML ES antigen may result in a false negative at the early stage of infection. The aim of the study was to characterize *T. spiralis* elastase-1 (TsEla) and to evaluate its potential as diagnostic antigen for trichinellosis.

**Methods:**

The complete cDNA sequences of the TsEla gene were cloned and expressed, and recombinant (rTsEla) was purified. TsEla transcription and expression in different *T. spiralis* life-cycle stages was investigated by qPCR and western blotting, and its location in the nematodes was evaluated using an immunofluorescence assay (IFA). The antigenicity of rTsEla was investigated by western blotting analysis and ELISA. Anti-*Trichinella* IgG, IgM and IgE of experimentally infected mice and specific IgG antibodies of trichinellosis patients were assayed by rTsEla-ELISA and ES-ELISA.

**Results:**

The results of the qPCR and western blotting showed that TsEla was expressed in various *T. spiralis* life stages. Natural TsEla was detected in the soluble proteins and ES proteins of different life stages. IFA revealed that TsEla was identified in the whole nematodes of various stages, especially in the cuticle, stichosome and genital primordium of the parasite. Serum anti-*Trichinella* IgM, IgG and IgE in infected mice was first detected by rTsEla-ELISA at 6, 10 and 12 days post-infection (dpi), and reached 100% at 8, 14 and 14 dpi, respectively. When rTsEla-ELISA and ES-ELISA were used to detect anti-*Trichinella* IgG in sera of trichinellosis patients, the sensitivity was 97.37% (37/38) and 89.74% (34/38) (*P* > 0.05), and the specificity was 99.10% (220/222) and 98.20% (218/222), respectively (*P* > 0.05). The rTsEla cross-reacted with only one serum sample out of 20 samples from paragonimiasis patients and 7 samples from clonorchiasis patients.

**Conclusions:**

rTsEla is valuable to early diagnosis of trichinellosis and could be an alternative diagnostic antigen to the ML ES antigens.
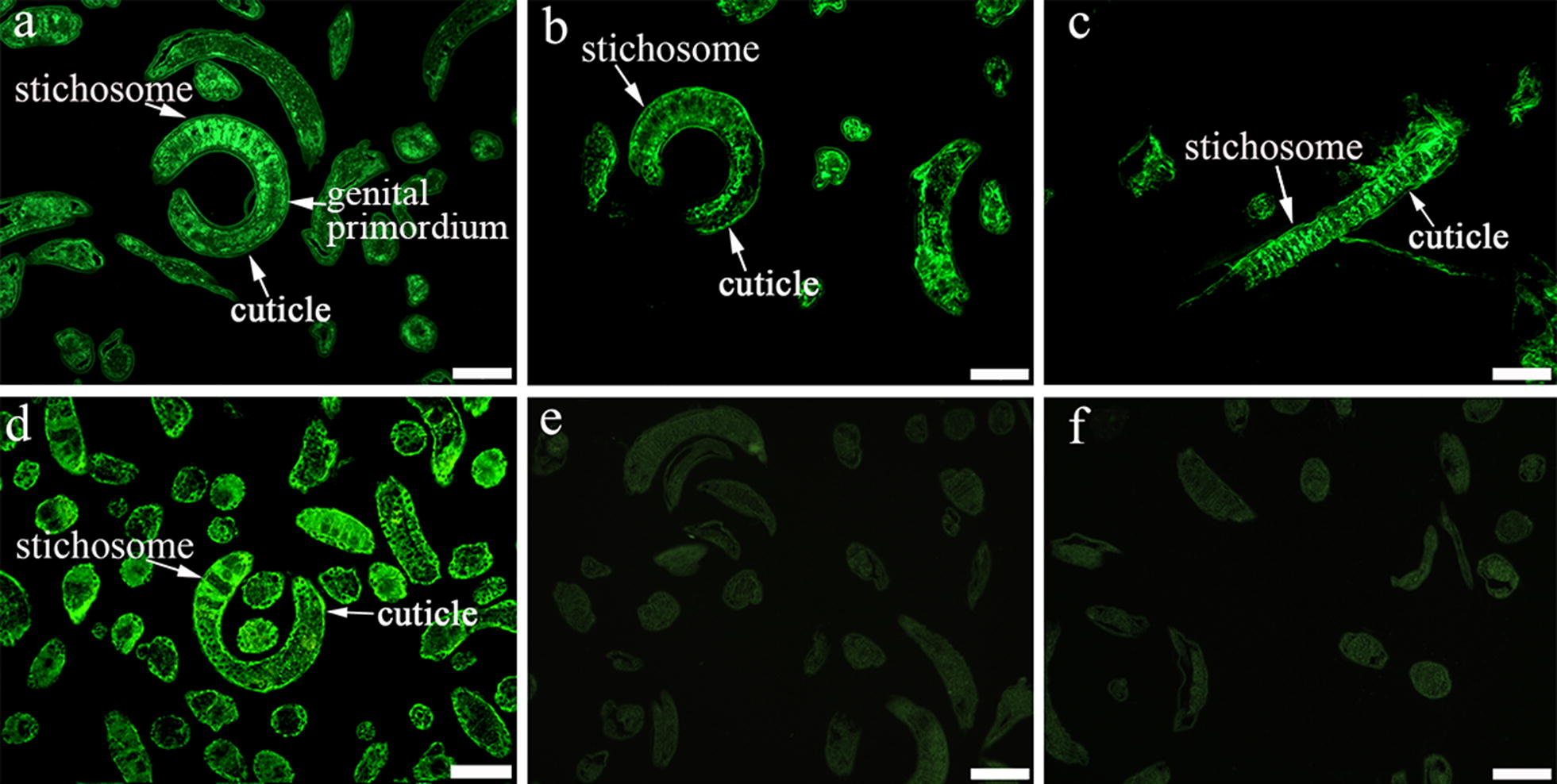

## Background

Trichinellosis is a globally distributed food-borne parasitic disease. Humans can become infected by ingesting raw or semi-cooked meat containing *Trichinella* muscle larvae (ML) [1]. Human *Trichinella* infection has been reported in 55 countries worldwide and trichinellosis is regarded to be a neglected zoonotic parasitosis. During 1986–2009, 65,818 cases and 42 deaths caused by trichinellosis were recorded in 41 countries [2, 3]. The main etiological agent of this disease is the common species *T. spiralis.* From 2004–2009, 15 trichinellosis outbreaks were documented in the Chinese mainland which consisted of 1387 cases and 4 deaths, with the dominating infection source being pork [4–6]. Trichinellosis has become an important food-borne zoonosis with direct risk for food safety and public health [7, 8].

After the infected animal meat is eaten, infectious *T. spiralis* muscle larvae are released from their capsules under the digestion of gastric fluid, they develop in the intestine into infectious larvae, which intrude intestinal epithelium and, after 4 molts, mature into the adult stage (AW) [9, 10]. The main symptom of the intestinal phase of trichinellosis is a non-specific gastroenteritis, with abdominal pain, diarrhea, nausea and/or vomiting, which may last up to 1 week. In 2–3 weeks after infection, each fertilized female deposits about 1500 newborn larvae (NBL), which migrate, penetrate and encapsulate in the host’s skeletal muscle (acute or muscle stage) [11]. The acute stage results in an inflammatory reaction in response to the larval penetration of the muscles, and its obvious manifestations are fever, eyelid/facial oedema, myalgia, and eosinophilia [12, 13].

Because of the non-specific manifestations, clinical diagnosis of trichinellosis is difficult [12]. Muscle biopsy or detection of specific IgG against *Trichinella* is the most commonly used definitive diagnostic method for human *Trichinella* infection, but examination of muscle biopsies for detection of the parasite is insufficiently sensitive to find *Trichinella* larvae in the early and mild stages of infection [14]. The International Commission on Trichinellosis (ICT) recommended an ELISA with ML excretion/secretion (ES) antigen to detect anti-*Trichinella* IgG [15], but the primary weakness of an muscle larval (ML) ES antigen is the potential of false-negatives due to specific IgG in the early stage of *T. spiralis* infection, which usually occurs in a 2–3 week window, because the major ML antigenic epitopes are stage-specific and can therefore only be probed by a ML stage-specific antibody [16]. The intestinal infective larvae (IIL) ES proteins are first exposed to the host’s enteral local mucosal and systematic immune system which triggers the generation of *Trichinella-*specific antibodies [17, 18]. The IIL ES proteins may contain early sero-diagnostic markers for *T. spiralis* infection [19]. Hence, the novel diagnostic antigens need to be exploited from the IIL ES proteins.

By immunoproteomics, several serine proteases have been identified in *T. spiralis* IIL and ML ES or surface proteins recognized using early infection serum [20–24]. Furthermore, *T. spiralis* serine proteases were highly expressed in IIL surface proteins relative to that of ML [25]. In this study, a novel *T. spiralis* elatase-1 gene (TsEla, GenBank: EFV56917.1) belonging to the serine protease family was obtained from the *T. spiralis* draft genome [26], cloned and expressed. Characteristics of the TsEla were investigated and the prospective serodiagnostic value of recombinant TsEla (rTsEla) for trichinellosis was evaluated using serum samples from infected animals and patients.

## Methods

### Parasites and experimental animals

*Trichinella spiralis* strain (ISS534), which was used in this study, was obtained from a domestic pig in central China [27]. An additional 4 species of *Trichinella* were gifted by the International *Trichinella* Reference Centre (ITRC, Rome, Italy): *T. nativa* (T2, ISS10); *T. britovi* (T3, ISS100); *T. nelsoni* (T7, ISS29); and *T. pseudospiralis* (T4, ISS13) [28]. Their passage was carried out in six-week-old female BALB/c mice which obtained from the Henan Provincial Experimental Animal Center (Zhengzhou, China).

### Worm collection and protein preparation

At 42 days post-infection (dpi), *T. spiralis-*infected mouse muscles were digested using 0.33% pepsin and 1% HCl to recover the ML [29, 30]. The IIL were collected from infected mouse intestine at 6 h post-infection (hpi) [25]. AW were acquired from duodenum and jejunum at 3 and 6 dpi, respectively [31]. Adult females were cultivated in RPMI-1640 medium containing 10% fetal bovine serum (FBS; Gibco, Thermo Fisher Scientific, Waltham, MA, USA) at 37 °C for 24 h, and then NBL were recovered [32]. The somatic crude or ES protein of NBL, ML, IIL and AW were prepared as previously described [33, 34].

### Serum samples

Fifteen mice were infected orally with 200 *T. spiralis* infective larvae. Following infection, 100 μl of tail blood was obtained on alternate days during 2–60 dpi [16, 35]. Serum samples collected at 35 dpi were prepared from mice infected with 300 larvae of five *Trichinella* species (*T. spiralis*, T1; *T. nativa*, T2; *T. britovi*, T3; *T. pseudospiralis*, T4 and *T. nelsoni*, T7). Sera from mice infected with *Toxoplasma gondii*, *Spirometra erinaceieuropaei* plerocercoids, *Schistosoma japonicum*, *Angiostrongylus cantonensis* and *Capillaria hepatica* were provided by parasitologists from various Chinese universities. Serum samples of uninfected mice were used as a negative control.

Thirty-eight trichinellosis serum samples were collected from 2 trichinellosis outbreaks that occurred in western China during 2003–2013 [36]. The serum of patients with hepatic capillariasis (*n* = 5), ancylostomiasis (*n* = 1), clonorchiasis (*n* = 7), paragonimiasis (*n* = 20), schistosomiasis (*n* = 30), sparganosis (*n* = 8), cysticercosis (*n* = 20) and echinococcosis (*n* = 20) were stored within our department. The definite diagnosis of these patients was made according to stool parasite examination or serological tests [37, 38]. Serum samples of 111 healthy individuals who were from non-endemic areas of trichinellosis and tested negative for the above-mentioned parasitoses, were also assayed in this work.

### Bioinformatics analysis of TsEla

The complete TsEla cDNA sequences were retrieved from GenBank (EFV56917.1). The characteristics of the TsEla gene were analyzed using bioanalysis software [39]. The tertiary structure and functional site of TsEla protein was predicted using PyMOL and CN3D software [40]. TsEla amino acid sequences were compared with elastases from other organisms by means of Clustal X [41]. The GenBank accession number of elastases from other organisms were as follows: *Trichinella nativa* KRZ58997.1; *T. britovi* KRY59723.1; *T. pseudospiralis* KRY01512.1; *Trichinella* sp. T6 KRX59470.1; *Trichinella* sp. T8 KRZ87740.1; *Trichinella* sp. T9 KRX64624.1; *T. murrelli* KRX35703.1; *T. nelsoni* KRX26142.1; *T. patagoniensis* KRY18991.1; *T. zimbabwensis* KRZ02345.1; *Ancylostoma duodenale* KIH49929.1; *Necator americanus* XP_013293254.1; *Ascaris suum* AEH42099.1; *Wuchereria bancrofti* EJW82097.1; *Brugia malayi* XP_001895362.1; *Caenorhabditis elegans* NP_494910.2; *Mus musculus* NP_291090.2; and *Homo sapiens* NP_001962.3. The maximum parsimony method was used to construct a phylogenetic tree of the elastase sequences using MEGA7.0 [42].

### Cloning and expression of rTsEla protein

Total RNA from *T. spiralis* ML was extracted using Trizol (Invitrogen, Carlsbad, CA, USA), and reversely transcribed to the first strand cDNA. The TsEla gene was amplified by PCR with specific primers carrying *BamH*I and *Pst*I restriction sites (underlined) (5′-CGG GAT CCT ATG AAT GTG GCA CCC TAC CAT CCA C-3′, 5′-GCG CTG CAG TTA ACG GAA AAA GGT GAA TGT TGG ATC ATT-3′). The PCR product was cloned into expression vector pQE-80L. The recombinant pQE-80L/TsEla was transformed into *Escherichia coli* BL21. Expression of rTsEla was induced at 37 °C for 6 h using 1.0 mM IPTG. rTsEla protein was purified using a Ni-NTA-Sefinose resin (Sangon Biotech, Shanghai, China) [43]. rTsEla proteins were analyzed on SDS-PAGE as previously described [44, 45].

### Preparation of polyclonal antibodies against rTsEla

Fifteen mice were immunized subcutaneously with 20 μg rTsEla mixed 1:1 (v/v) with complete Freund’s adjuvant. Three booster immunizations were administered using the same dose of rTsEla mixed with incomplete Freund’s adjuvant at a two-week interval [46]. Two weeks after the final boost, 100 μl of tail blood was taken from immunized mice, and anti-rTsEla serum was isolated [47].

### Western blotting analysis

Somatic soluble proteins of *T. spiralis* different life stages and rTsEla were separated on SDS-PAGE, and transferred onto nitrocellulose membranes (Merck Millipore, Billerica, MA, USA) at 18 V for 35 min [48]. The membrane was cut into strips, and blocked using 5% skimmed milk at 37 °C for 2 h. After washing with TBS-0.5% Tween 20 (TBST), the strips were probed with anti-rTsEla serum (1:100) for 2 h at 37 °C, followed by incubation with HRP-anti-mouse IgG conjugate (1:5000; Southern Biotech, Tuscaloosa, AL, USA) at 37 °C for 1 h. Following washing again, the strips were developed using 3,3′-diaminobenzidine tetrahydrochloride (DAB; Sigma-Aldrich, St. Louis, MO, USA) [31, 49].

### qPCR

Total RNA of different *T. spiralis* life stages (NBL, ML, IIL, 3 d AW and 6 d AW) was extracted using Trizol reagent (Invitrogen). TsEla transcription levels were detected by qPCR with specific primers (5′-TGG AAA AAG ATG GTA ATA GAA TA A A-3′; 5′-TGT TAT CAA AAT CTG CAT AGT AGG T-3′) as previously reported [50, 51]. *T. spiralis* internal control gene GAPDH (GenBank: AF452239) was amplified as a positive control [52]. PBS served as a negative control. TsEla transcription level was standardized by deducting the GAPDH transcription level and then calculated by comparative Cq (2^−ΔΔCq^) [53].

### Immunofluorescence assay (IFA)

To locate TsEla in worm tissues, IFA was performed with *T. spiralis* whole worms and their cross-sections [54, 55]. The integrated parasite was fixed in cold acetone for 20 min and 2 μm worm cross-sections were cut using a microtome. The whole worms and cross-sections were blocked with 1% bovine serum albumin (BSA) and probed by anti-rTsEla serum diluted at 1:10 at 37 °C for 2 h. After washing with PBS, they were incubated at 37 °C for 1 h with cy3/FITC anti-mouse IgG conjugate (1:100; Santa Cruz Biotech, Dallas, Texas, USA), and examined using fluorescence microscopy (Olympus, Tokyo, Japan) [56].

### Application of rTsEla-ELISA to assay *Trichinella-*specific antibodies

Anti-T*richinella* IgG, IgM and IgE of experimentally infected mice and specific IgG antibodies of trichinellosis patients were assayed by ELISA with rTsEla (rTsEla-ELISA) and ML ES antigens (ES-ELISA) [57, 58]. Optimal dilutions of antigens and sera were first determined using a checkerboard titration. The optimal antigen coating concentration was 2.0 μg/ml rTsEla for detecting specific IgG and IgM, 3.5 μg/ml rTsEla for detecting IgE, and 2.5 μg/ml ES antigens for IgG, IgM and IgE. Microtiter plates were coated with the above mentioned antigens, and blocked using 5% skimmed milk at 37 °C for 2 h. After washes with PBST, the plates were probed with mouse sera (1:100 for IgG and 1:50 for IgM and IgE), and human sera diluted at 1:200 for detecting three antibodies, subsequently incubated at 37 °C for 1 h with HRP-labelled anti-human/mouse IgG antibody (1:10,000; Southern Biotech). Following washes, the plates were developed using o-phenylenediamine dihydrochloride (OPD; Sigma-Aldrich) with 30% H_2_O_2_ for 30 min, and the reaction was stopped using 2M H_2_SO_4_. The absorbance (optical density, OD) at 492 nm was assayed with a microplate reader (TECAN, Schweiz, AG, Switzerland), and all serum samples were tested in duplicate. The ratio < 2.1 of tested serum/negative serum OD values was considered as negative and the ratio ≥ 2.1 as positive [59, 60]. For detecting mouse serum IgG, IgM and IgE, the cut-off values for rTsEla-ELISA were 0.25, 0.24 and 0.21, respectively; and those for ES-ELISA were 0.29, 0.27 and 0.23, respectively. The cut-off values of rTsEla-ELISA and ES-ELISA for detecting human IgG were 0.34 and 0.61, respectively.

### Statistical analysis

SPSS 21.0 software was used to statistically analyze the data. Data are shown as the arithmetic mean ± standard deviation (SD). TsEla transcription and expression in different *T. spiralis* life stages were compared using one-way ANOVA. The difference between groups was determined by Chi-square test. A statistical difference was considered significant at *P* < 0.05.

## Results

### Bioinformatics analysis of TsEla

**Fig. 1:**
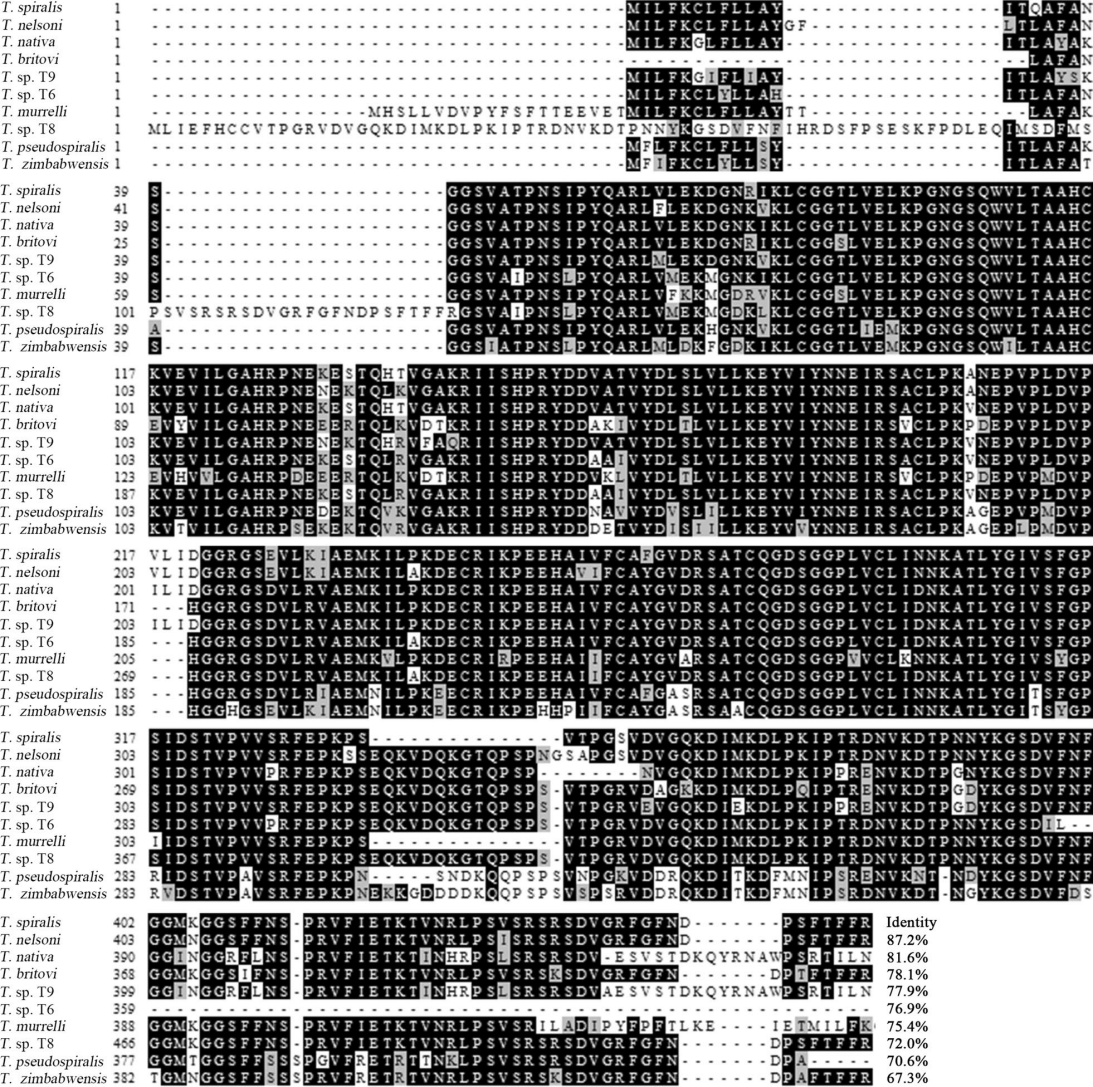
Sequence alignment of *Trichinella spiralis* elastase gene (XP_003377838.1) with other *Trichinella* species or genotypes. Clustal X and BOXSHADE were used to analyze the sequences, obvious differences were observed in different species/genotypes. Black shading represents the residues identical to TsEla, and grey shading shows the conservative substitutions

Complete TsEla coding sequence consisted of 1350 bp encoding 449 amino acids (aa), with a molecular weight (MW) of 47.3 kDa and isoelectric point (pI) of 8.88. A signal peptide at 19 aa was detected by Signal P 4.1 Server. TMHMM prediction revealed that TsEla has no transmembrane domain, but has a functional domain named as Tryp_SPc located at 38–314 aa. The TsEla homology comparison of the TsEla gene with those of other species or genotypes of *Trichinella* is shown in Fig. 1. The TsEla amino acid sequence had an identity of 87.2, 81.6, 78.1, 77.9, 77.6, 75.4 and 72.0% with elastase of the 7 species with encapsulated larvae (*T. nelsoni*, *T. nativa*, *T. britovi*, T9, T6, *T. murrelli* and T8), and an identity of 70.6 and 67.3% with species with non-encapsulated larvae (*T. pseudospiralis* and *T. zimbabwensis*, respectively).

**Fig. 2:**
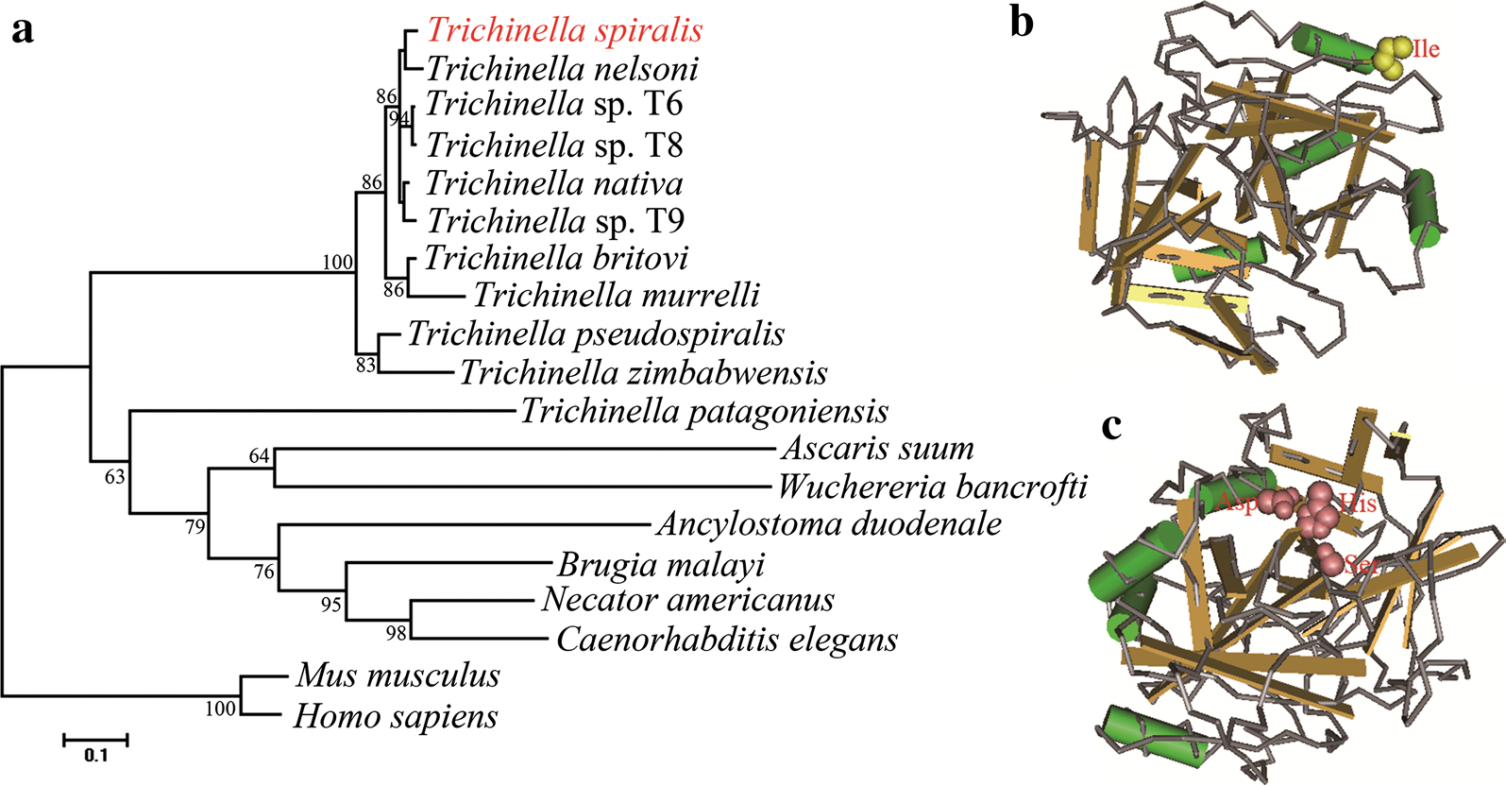
Phylogenetic tree of elastases of 19 organisms generated with MP method (**a**) and the predicted 3-dimensional structure of TsEla protein (**b**, **c**). **b** The predicted catalytic site Ile of the TsEla (in yellow). **c** Three serine protease-specific active sites (His, Asp and Ser) of TsEla, which are marked as red

Phylogenetic analysis of TsEla with elastase-1 from other nematodes is shown in Fig. 2a. The phylogenetic tree shows the monophyletic group of above-mentioned 10 *Trichinella* species/gene types, except for the recently described *T. patagoniensis* from Argentina. According to the phylogenetic analysis of elastase, *T. spiralis* has a closer evolutionary relationship with the species of the genus with encapsulated larvae. The structure prediction revealed that TsEla has 5 α-helices and 37 β-strands, a catalytic site Ile, and three serine protease-specific active sites (His, Asp and Ser) (Fig. 2b, c).

### SDS-PAGE and western blotting analysis of rTsEla

**Fig. 3:**
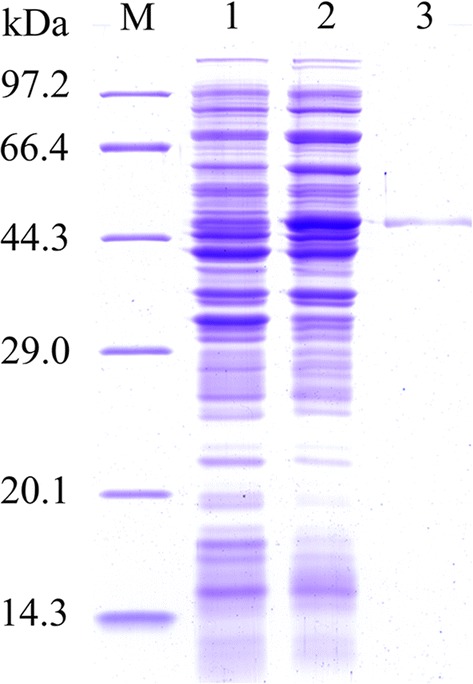
SDS-PAGE analysis of rTsEla. Lane M: protein marker; Lane 1: lysate of BL21 bacteria containing pQE-80L/TsEla before induction; Lane 2: lysate of BL21 bacteria containing pQE-80L/TsEla following induction; Lane 3: rTsEla after purification

**Fig. 4:**
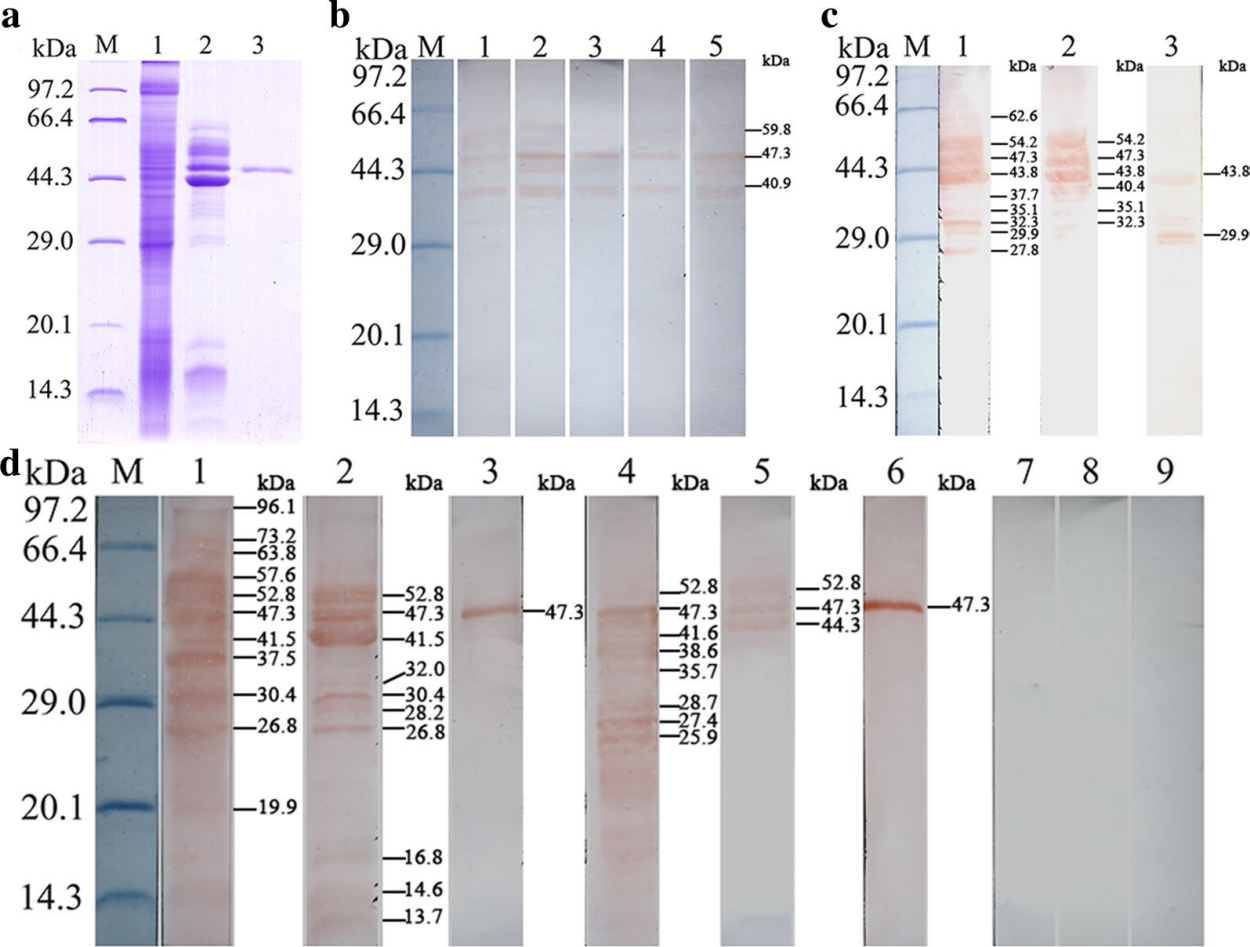
Western blotting identification of rTsEla. **a** SDS-PAGE analysis of rTsEla. Lane M: protein marker; Lane 1: ML soluble proteins; Lane 2: ML ES proteins; Lane 3: purified rTsEla. **b** Western blotting revealed natural TsEla in soluble proteins of *T. spiralis* NBL (Lane 1), ML (Lane 2), IIL (Lane 3), 3d AW (Lane 4) and 6d AW (Lane 5) were probed by anti-rTsEla serum. **c** Western blotting showed that native TsEla in ES proteins of ML (Lane 1), IIL (Lane 2) and 3 d AW (Lane 3) were detected by anti-rTsEla serum. **d** Western blotting of rTsEla antigenicity. ML soluble protein (Lane 1), ML ES protein (Lane 2) and rTsEla (Lane3) were probed by *T. spiralis*-infected mouse sera as positive serum control; native TsEla in ML soluble (Lane 4) and ES protein (Lane 5), and rTsEla (Lane 6) were recognized by anti-rTsEla serum; ML soluble (Lane 7) and ES protein (Lane 8), and rTsEla (Lane 9) were not probed by uninfected mouse serum as negative serum control

SDS-PAGE revealed that BL21 bacteria incorporating PQE-80L/TsEla, expressed a 47.3 kDa fusion protein. After purification, the rTsEla protein exhibited a clear individual band (Figs. 3, 4d). The MW (47.3 kDa) of the rTsEla protein was identical with its predicted MW size (47.3 kDa).

To assess the murine humoral response to rTsEla, anti-rTsEla IgG antibody in immunized mice was measured by ELISA. The results indicated anti-rTsEla IgG was induced by immunization with rTsEla. The specific IgG titer was up to 1:10^5^ after the fourth immunization, demonstrating that the rTsEla has good immunogenicity. Western blotting revealed that three bands of native TsEla with 40.9–59.8 kDa in soluble protein of different *T. spiralis* life stages (NBL, ML, IIL, 3d and 6d AW) were detected by anti-rTsEla serum (Fig. 4b), 2–9 bands of native TsEla with 27.8–62.6 kDa in ML, IIL and 3d AW ES proteins was also probed using anti-rTsEla serum (Fig. 4c). Eight bands of native TsEla with 25.9–52.8 kDa in ML soluble proteins, three bands of native TsEla with 44.3–52.8 kDa in ML ES proteins, and rTsEla were detected by anti-rTsEla serum, but not by non-infected serum (Fig. 4d), indicating that TsEla was expressed at diverse *T. spiralis* stages, and it is a secretory protein of this nematode.

### qPCR analysis of TsEla gene transcription in different life stages of *T. spiralis*

**Fig. 5:**
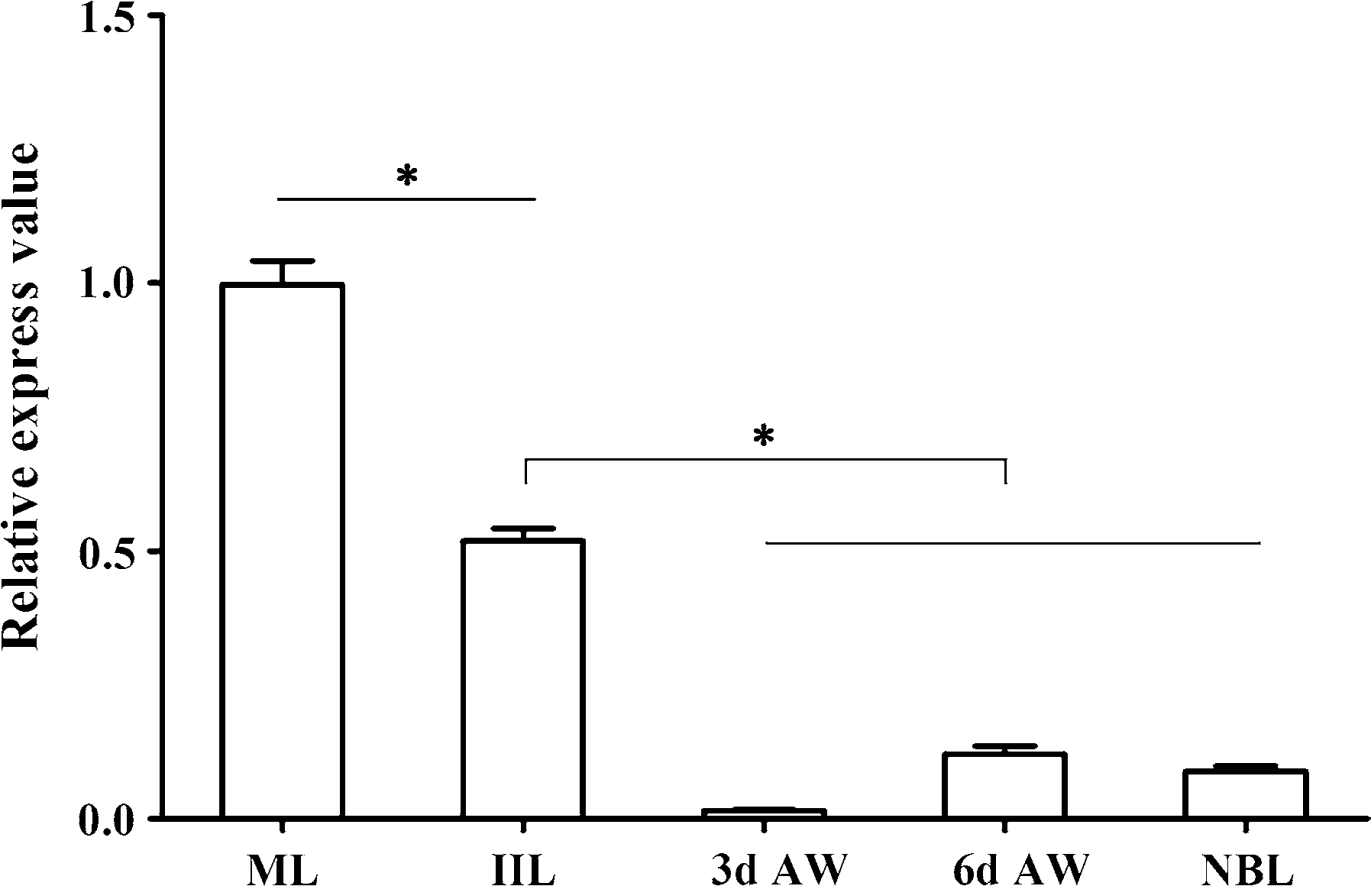
qPCR analysis of TsEla transcription in different *T. spiralis* stages. The transcription levels of TsEla were calculated with the Cq (2^−ΔΔCq^) method. GAPDH was utilized as an internal control. *P < 0.0001

Transcription of the TsEla gene in different life stages of *T. spiralis* was detected by qPCR, the results revealed that the TsEla gene was transcribed at all *T. spiralis* stages (NBL, ML, IIL, 3 d and 6 d AW) (Fig. 5). The transcription level in the ML stage was obviously higher than those of the other stages (*F*_(4, 10)_ = 287.184, *P* < 0.001), and the TsEla level in the IIL stage was higher than those in the NBL and AW stage (*P* < 0.0001), but lower than that in the ML stage (*P* < 0.0001).

### Expression and localization of TsEla in different *T. spiralis* stages by IFA

**Fig. 6:**
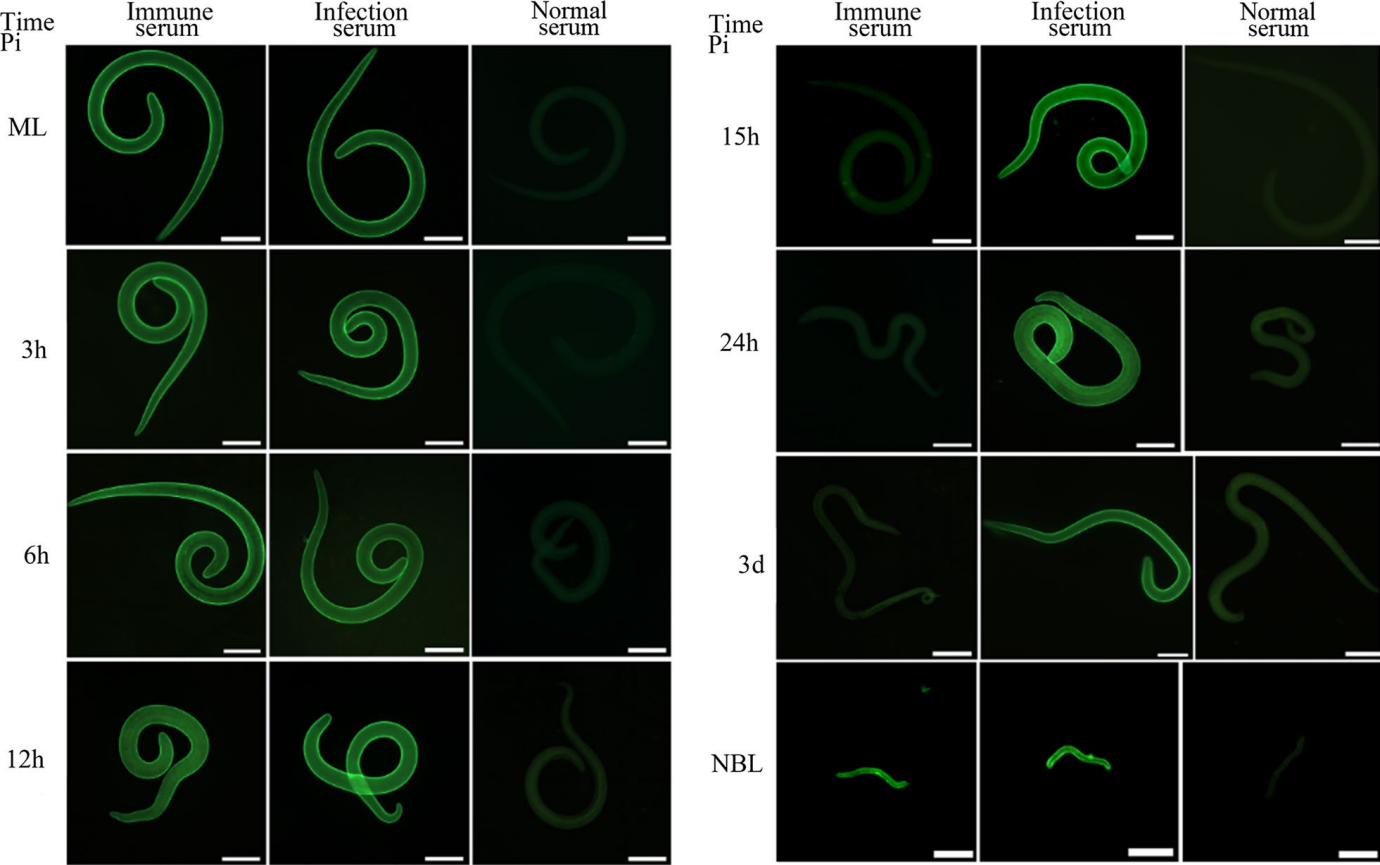
Expression of TsEla at the surface of different *T. spiralis* stages by IFA with anti-rTsEla serum. Fluorescence staining was found at the cuticle of ML, 3–12 h IIL and NBL by using anti-rTsEla serum, but no immunostaining was found in the cuticle of 15–24 h IIL and 3 d AW. The worms reacted with infected or uninfected serum as a positive or negative control. *Scale*-*bars*: 100 µm

**Fig. 7:**
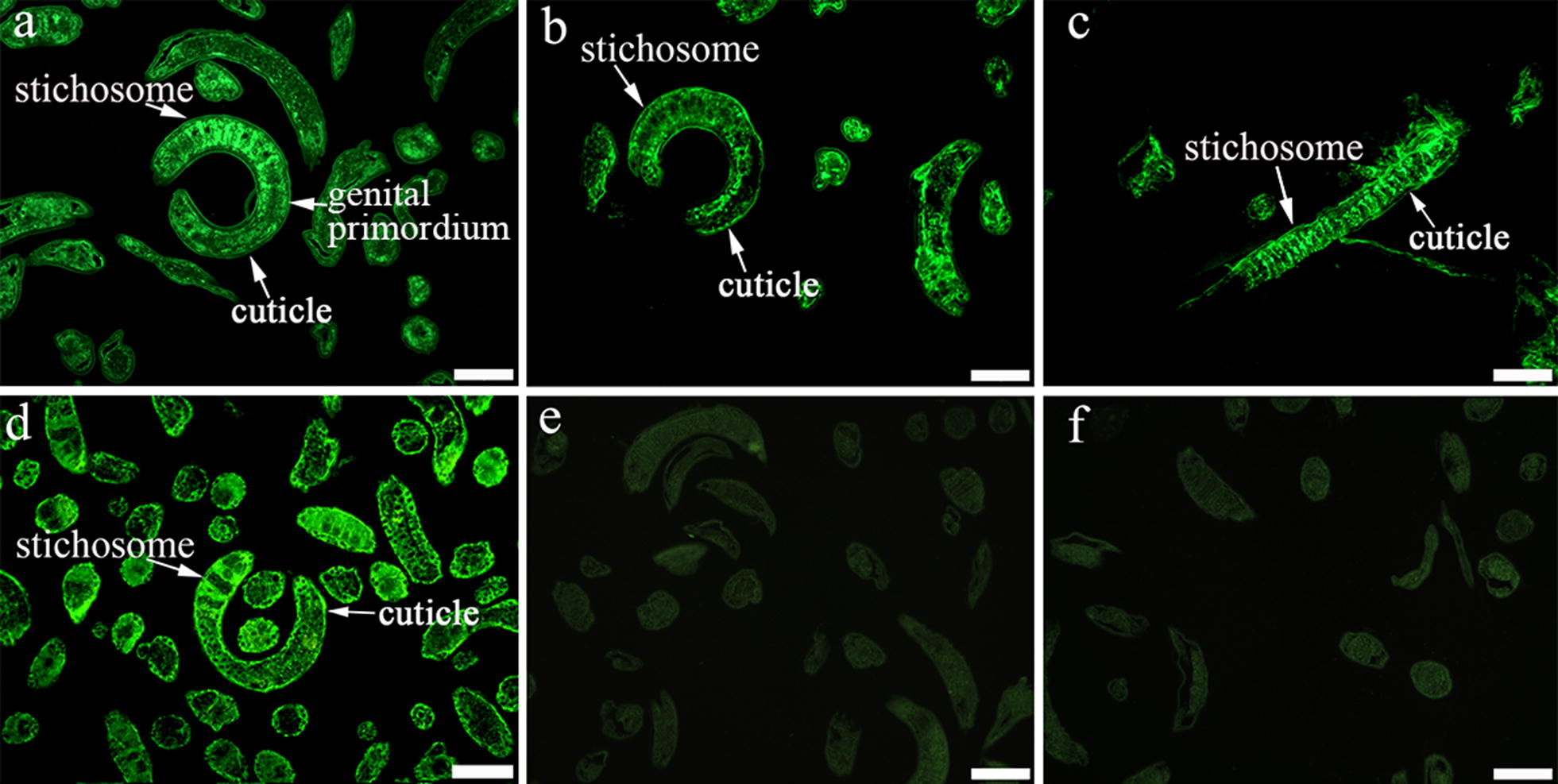
Immunolocalization of TsEla in cross-sections of different *T. spiralis* life stages by IFA with anti-rTsEla serum. The parasite cross-sections were probed using anti-rTsEla serum, and fluorescence staining was seen in the cuticle and stichosome of ML (**a**), 6 h IIL (**b**) and 3 d AW (**c**). ML cross-sections were incubated with infection sera (**d**) as a positive control, uninfected sera (**e**) and PBS (**f**) as negative controls. *Scale*-*bars*: 100 µm

IFA was used to detect the expression and localization of TsEla in *T. spiralis* stages. IFA with entire worms detected positive fluorescence staining in the epicuticle of ML, early IIL (3, 6 and 12 h IIL) and NBL by anti-rTsEla serum, but no immunostaining was found in late IIL (15 and 24 h IIL) and 3 d AW (Fig. 6), suggesting that native TsEla was expressed in the epicuticle of ML, early IIL and NBL, but not in the epicuticle of late IIL and AW stages. When worm cross-sections were used in IFA, fluorescence staining was distributed within the whole body in ML, 6 h IIL and 3 d AW, especially in the cuticle, stichosome and genital primordium of the parasites (Fig. 7). No fluorescence staining in ML was observed by incubation with mouse pre-infected serum.

### Assay of *Trichinella-*specific IgG in sera of mice infected with *Trichinella* and other parasites

*Trichinella*-specific IgG in serum samples from mice infected with *T. spiralis* and other parasites was tested using rTsEla-ELISA and ES-ELISA; the result is shown in Table 1. The detection rate of specific IgG using two antigens was 100% of mice infected with 300 *T. spiralis* ML at 35 dpi. Both antigens did not cross-react with sera from mice infected with *T. gondii*, *Capillaria hepatica*, *A. cantonensis*, *Clonorchis sinensis* and *S. japonicum.* However, ML ES antigen had a cross-reactivity with one serum (4.8%) from sparganum-infected mice. Specific IgG in serum from mice infected with 300 larvae of other *Trichinella* species were also measured using two antigens (Table 2). The IgG detection rate with two antigens had also no significant difference in mice infected with the other four *Trichinella* species (${\chi^2}_{\left( 1 \right)}$ = 0.317, *P* = 0.573).

**Table 1:** Detection of serum anti-*Trichinella* IgG in mice infected with *Trichinella spiralis* and other parasites by rTsE-ELISA and ES-ELISA

Parasite species	No. of serum samples	ELISA with rTsE		ELISA with ML ES	
		OD ± SD	No. of positive samples (%)	OD ± SD	No. of positive samples (%)
*Trichinella spiralis*	20	1.24 ± 0.72	20 (100)	1.32 ± 0.77	20 (100)
*Capillaria hepatica*	10	0.05 ± 0.03	0 (0)	0.06 ± 0.03	0 (0)
*Angiostrongylus cantonensis*	12	0.05 ± 0.02	0 (0)	0.05 ± 0.01	0 (0)
*Clonorchis sinensis*	20	0.11 ± 0.05	0 (0)	0.13 ± 0.06	0 (0)
*Schistosoma japonicum*	16	0.09 ± 0.05	0 (0)	0.11 ± 0.06	0 (0)
*Spirometra erinacei*	21	0.12 ± 0.01	0 (0)	0.16 ± 0.06	1 (4.8)
*Toxoplasma gondii*	6	0.07 ± 0.05	0 (0)	0.08 ± 0.05	0 (0)

*Abbreviations*: OD, OD-value; SD, standard deviation

**Table 2:** Detection of serum anti-*Trichinella* IgG in mice infected with four *Trichinella* species by ELISA provide the same detail as in Table 1

Parasite species	No. of serum samples	ELISA with rTsE		ELISA with ML ES	
		OD ± SD	No. of positive samples (%)	OD ± SD	No. of positive samples (%)
*T. nativa*	23	0.48 ± 0.31	21 (91.3)	0.51 ± 0.34	21 (91.3)
*T. britovi*	14	0.71 ± 0.36	14 (100)	0.68 ± 0.38	14 (100)
*T. pseudospiralis*	30	0.39 ± 0.23	22 (73.3)	0.4 ± 0.24	20 (66.7)
*T. nelsoni*	15	0.75 ± 0.47	14 (93.3)	0.72 ± 0.47	14 (93.3)

*Abbreviations*: OD, OD-value; SD, standard deviation

### Dynamics of serum *Trichinella*-specific IgG, IgM and IgE in mice infected with 200 larvae

**Fig. 8:**
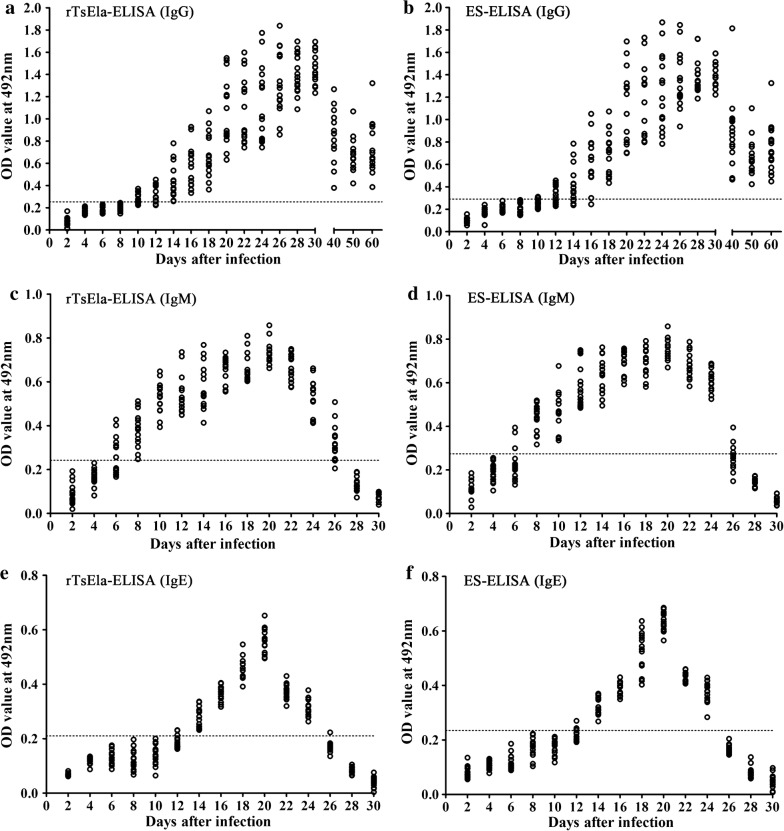
Kinetics of serum *Trichinella*-specific antibodies in mice infected with 200 *T. spiralis* larvae. Specific IgG was measured with rTsEla-ELISA (**a**) and ES-ELISA (**b**); anti-*Trichinella* IgM by rTsEla-ELISA (**c**) and ES-ELISA (**d**), and anti-*Trichinella* IgE by rTsEla-ELISA (**e**) and ES-ELISA (**f**). The dotted lines represent the cut-off values

At 10 dpi, anti-*Trichinella* IgG was primarily detected in 73.33% (11/15) of infected mice by rTsEla-ELISA and in 20% (3/15) by ES-ELISA (${\chi^2}_{\left( 1 \right)}$ = 8.571, *P* = 0.003). Seroconversion reached 100% (15/15) at 14 and 18 dpi determined by two antigens, respectively (Fig. 8a, b), and remained as this until the end of the experiment (60 dpi). When anti-*Trichinella* IgM was assayed, it was first detected at 6 dpi in 40% (6/15) of infected mice by rTsEla-ELISA and in 20% (3/15) by ES-ELISA (${\chi^2}_{\left( 1 \right)}$ = 1.429, *P* = 0.232), and the seroconversion reached the peak (100%) at 8 dpi, persisted to 24 dpi, but was not detected by 28 dpi (Fig. 8c, d). When anti-*Trichinella* IgE was measured by two antigens, it was first detected at 12 dpi in 20% and in 26.7% of infected mice (${\chi^2}_{\left( 1 \right)}$ = 0.186, *P* = 0.666), respectively; antibody positivity reached 100% at 14 dpi, and became negative by 28 and 26 dpi, respectively (Fig. 8e, f).

### Assay of *Trichinella*-specific IgG in serum of trichinellosis patients

**Fig. 9:**
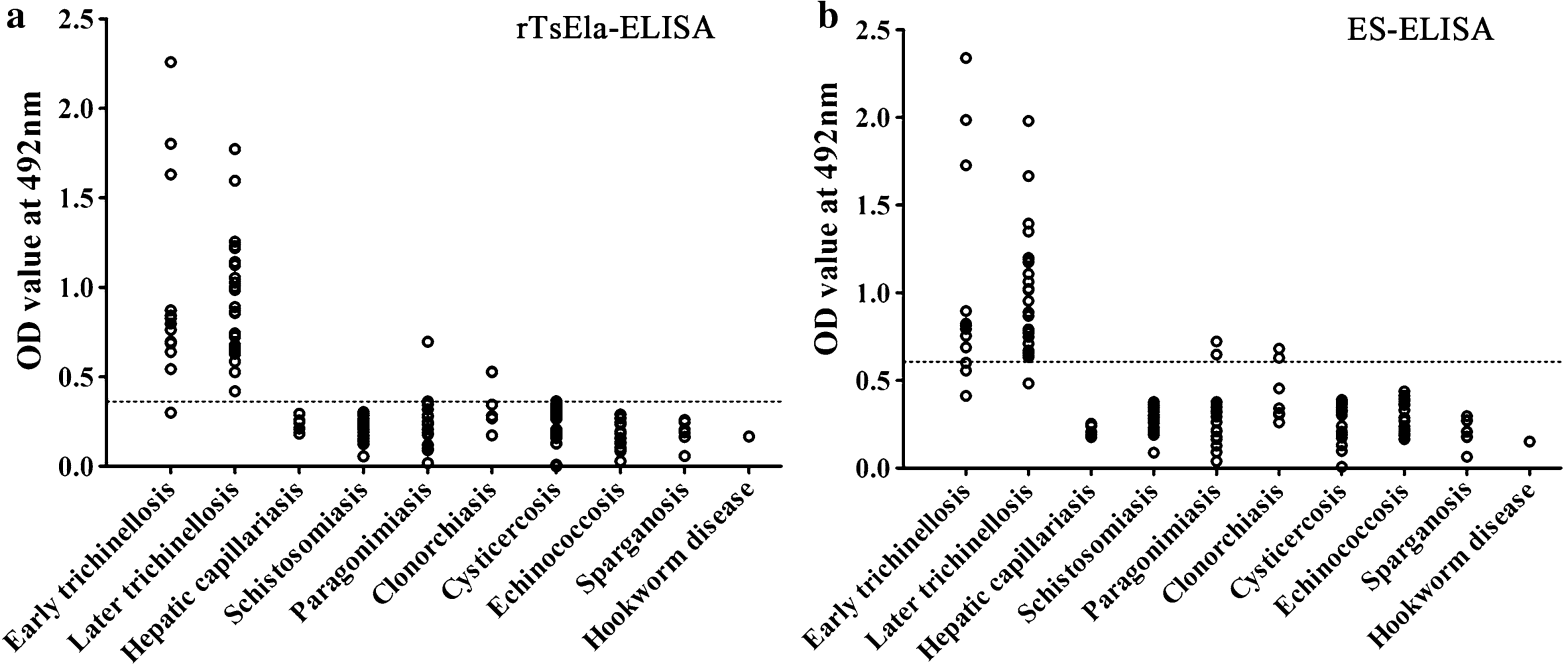
Scatter diagram of OD values of rTsEla-ELISA (**a**) and ES-ELISA (**b**) for detecting serum anti-*Trichinella* IgG in patients with trichinellosis and other parasitoses. The cut-off values are shown as a dotted line

Sensitivity of rTsEla-ELISA and ES-ELISA for assaying *Trichinella*-specific IgG in serum from trichinellosis patients was 97.37% (37/38) and 89.74% (34/38), respectively (${\chi^2}_{\left( 1 \right)}$ = 1.020, *P* = 0.312). When the patients sera were tested at 19 dpi, the sensitivity of rTsEla was 92.31% (12/13), which had no statistical difference compared with 76.92% (10/13) for the ES antigen ($\chi^{2}_{\left( 1 \right)}$ = 1.927, *P* = 0.165) (Table 3). The specificity of rTsEla and ES antigen was 99.10% (220/222) and 98.20% (218/222) (${\chi^2}_{\left( 1 \right)}$ = 0.676, *P* = 0.411), when they were used to assay anti-*Trichinella* IgG in patients with other parasitoses and healthy individuals. No cross-reaction of rTsEla was observed with serum samples of patients with hepatic capillariasis, ancylostomiasis, schistosomiasis, cysticercosis, sparganosis, and echinococcosis, but rTsEla did cross-react with one serum from a patient with paragonimiasis and another with clonorchiasis (Fig. 9).

**Table 3:** Detection of anti-*Trichinella* IgG antibodies in serum samples of patients with trichinellosis and other parasitoses by rTsE-ELISA and ES-ELISA

Sera of mice infected with specific parasites	No. of serum samples	ELISA with rTsE		ELISA with ML ES	
		OD ± SD	No. of positive samples (%)	OD ± SD	No. of positive samples (%)
Trichinellosis	38	0.95 ± 0.41	37 (97.37)	1.00 ± 0.44	34 (89.47)
Early trichinellosis^a^	13	0.97 ± 0.54	12 (92.31)	1.00 ± 0.58	10 (76.92)
Late trichinellosis^b^	25	0.94 ± 0.32	25 (100)	1.00 ± 0.34	24 (96.00)
Hepatic capillariasis	5	0.24 ± 0.04	0 (0)	0.21 ± 0.03	0 (0)
Ancylostomiasis	1	0.167	0 (0)	0.152	0 (0)
Paragonimiasis	20	0.27 ± 0.14	1 (5.00)	0.28 ± 0.16	2 (10.00)
Schistosomiasis	30	0.21 ± 0.06	0 (0)	0.28 ± 0.07	0 (0)
Clonorchiasis	7	0.34 ± 0.13	1 (14.29)	0.41 ± 0.13	2 (28.57)
Cysticercosis	20	0.24 ± 0.11	0 (0)	0.27 ± 0.11	0 (0)
Echinococcosis	20	0.18 ± 0.10	0 (0)	0.30 ± 0.10	0 (0)
Sparganosis	8	0.20 ± 0.06	0 (0)	0.21 ± 0.07	0 (0)
Healthy individuals	111	0.16 ± 0.06	0 (0)	0.29 ± 0.04	0 (0)

^a^Early trichinellosis, the sera of patients with early trichinellosis was collected at 19 days after infection

^b^Late trichinellosis, the sera of patients with late trichinellosis was collected at 35 days after infection

*Abbreviations*: OD, OD-value; SD, standard deviation

## Discussion

Detection of circulating antigen or DNA from live *Trichinella* worms is an ideal early diagnostic technique for detecting *Trichinella* infection. However, the content of *Trichinella* circulating antigen and DNA in serum samples is usually low and difficult to detect [16, 61]. In addition, the easy degrading and short duration of *Trichinell*a DNA leads to its non-application as a diagnostic marker for *Trichinella* infection [62, 63]. Hence, novel diagnostic antigens for early *Trichinella* infection need to be explored.

Elastases are the trypsin-like proteins belonging to the family of serine proteases, they can degrade host’s various macromolecular substances such as keratin, laminin, type IV collagen, fibronectin and elastin [64]. The elastases are involved in larva invasion, molting and digestion, and may exert a major action for parasite invasion [65, 66]. Elastases are likely significant target antigens for eliciting an immune response at an early stage of infection, and may be used as diagnostic markers for early parasitic diseases. Nevertheless, studies on elastases were mainly focused on *Schistosoma* spp. [67, 68]; we found no reports of studies on *T. spiralis* elastase in the literature.

In the present study, the complete TsEla sequence was cloned and expressed in *E. coli*. Bioinformatics analysis showed that the TsEla had a signal peptide and one functional domain of three serine protease-specific active sites. Sequence analysis showed that TsEla has 87.2% and 81.6% identity with the elastase of *T. nelsoni* and *T. nativa*, respectively. The phylogenetic tree indicated TsEla had a close evolutionary relationship with elastase of *T. nelsoni,* and demonstrated a monophyletic group of 10 *Trichinella* species/gene types, except for *T. patagoniensis* which was recently found in a cougar from Argentina [69]. Following purification, the rTsEla was highly immunogenic and used to produce anti-rTsEla serum. Immunization of mice with rTsEla triggered the obvious antibody responses to rTsEla. qPCR revealed that TsEla transcription was detected in various *T. spiralis* stages (ML, IIL, AW and NBL), indicating that TsEla was transcribed at every life-cycle stage of this nematode, and the TsEla transcription level at ML and IIL stages was remarkably greater than that of other stages as shown in Fig. 5. Western blotting showed that several native TsEla protein bands in ML somatic and ES proteins were identified by anti-rTsEla serum, it is likely because TsEla might have various isoforms, or this protein was possibly processed by post-translational modifications and processing, or because TsEla is one member of the *Trichinella* serine protease superfamily and possesses the same antigenic epitopes [13, 36, 70].

Western blotting results also revealed that native TsEla in ML, IIL and 3 d AW ES proteins were recognized by anti-rTsEla serum. The results of IFA with intact worms showed that native TsEla was mainly expressed in the epicuticle of ML, early IIL (3–12 h IIL) and NBL, but not in late IIL (15–24 h IIL) and AW. The native TsEla was distributed in the whole worms of ML, 6 h IIL and 3 d AW, especially in the cuticle, stichosome and genital primordium of the parasite when worm cross-sections were used. Concerning the TsEla location, some differences in 3 d adults occurred between Figs. 6 and 7c, it is likely because when whole worms were used in the IFA, their surface cuticle was intact, and the inner antigens were not exposed, and not recognized by anti-rTsEla serum. Whereas when the cross-sections of 3 d adult worms were prepared, the worm surface cuticle (epicuticle) might be destroyed by the hot paraffin in the process of worm embedding, so the inner antigens were exposed and therefore recognized and immuno-stained. These results suggest that TsEla is a secretory protein which is likely derived from surface/ES protein of this nematode. Previous studies have demonstrated that other *T. spiralis* serine proteases (TspSP-1.2, TsSP and Ts31) were also expressed in the cuticle and ES products of the parasitic nematode [13, 36, 48], suggesting that TsEla is an indispensable protein of parasite-host interaction, and might have an important action in larva invasion and eliciting an early immune response. However, the rTsEla enzyme activity and functions need to be further investigated.

In order to evaluate the rTsEla potential for serodiagnosis of trichinellosis, an rTsEla-ELISA was established, and the results were comparatively analyzed with those of a conventional ML ES-ELISA. Our results revealed that anti-*Trichinella* IgM, IgG and IgE in infected mice were first detected by rTsEla-ELISA at 6, 10, 12 dpi, and reached 100% at 8, 14 and 14 dpi, respectively. When the ES-ELISA was used, anti-*Trichinella* IgM, IgG and IgE in infected mice were first detected at 6, 10, 12 dpi, and reached 100% at 8, 18 and 14 dpi, respectively, indicating that anti-*Trichinella* IgM levels rapidly elevated during the early phase of *Trichinella* infection, but declined quickly from the beginning of 26 dpi when the larvae were encapsulated in the skeletal muscles [11]. The results suggest that detection of anti-*Trichinella* IgM is valuable only for early trichinellosis [16, 71]. Because of the short half-life in serum samples, IgE had no advantage to conventional IgG detection [12, 72]. The amount and avidity of IgG antibodies was found to be most useful for serodiagnosis of trichinellosis [73], the seroconversion time following primary *Trichinella* infection was dependent on the infectious dose and muscle larva burden, anti*-Trichinella* IgG was usually detected approximately 2–3 weeks post-infection in infected animals, and may remain for many years [74].

Interestingly, *Trichinella*-specific IgG in 100% of mice infected with 200 *T. spiralis* larvae were detected using rTsEla-ELISA as early as 14 dpi, but the ES-ELISA could not detect all of the infected mice prior to 18 dpi. Our results suggest that TsEla as a surface/ES protein of ML and early IIL was exposed with host’s immune system at an early stage of the infection, triggering an early specific IgG response which continued to the muscle stage [17, 25].

In order to ascertain the sensitivity and specificity of rTsEla for detecting anti*-Trichinella* IgG, rTsEla-ELISA was used to test serum samples from patients with trichinellosis and other parasitoses, and the results were compared with ES-ELISA. The results showed that the sensitivity of rTsEla-ELISA and ES-ELISA was 97.37% and 89.47%, respectively (*P* > 0.05). When the serum samples from patients at the early stage of trichinellosis at 19 dpi were examined, the sensitivity of rTsEl-ELISA (92.31%) was higher than 76.92% of ES-ELISA, but there was no statistically significant difference (*P* > 0.05); this result is likely because only a few of the early serum samples were assayed. Specificity of rTsEla and ES antigens was 99.10% and 98.20%, respectively (*P* > 0.05), when the two antigens were used to test *Trichinella-*specific IgG in sera from patients with other helminthiases and healthy individuals. rTsEla cross-reacted with only one out of 20 paragonimiasis patients and seven clonorchiasis patients. Because of the long-time storage and repeated freezing/thawing of these serum samples, anti-*Trichinella* IgM in our patients’ sera were not detected (data not shown). Sensitivity and specificity of rTsEla are comparative to those of recombinant Ts31 and TsSP proteins [13, 36]. Moreover, preparation of ML ES antigens requires collecting the infectious larvae from experimentally infected animals, which is inconvenient due to labor, time and costs [32]. Recombinant antigens can be produced *in vitro* in large quantities and may be used as a diagnostic antigen in a standardized ELISA to diagnose trichinellosis [58]. Therefore, the rTsEla is worthy of early serodiagnosis of trichinellosis, and could be useful as a potential alternative diagnostic antigen to the ML ES antigens.

## Conclusions

Our study indicates that TsEla was transcribed and expressed in all life-cycle stages of *T. spiralis*, it was likely derived from the nematodeʼs surface and ES proteins, and located in the whole worm body. The rTsEla had a good immunogenicity. The sensitivity and specificity of rTsEla for assaying *Trichinella-*specific IgG and IgM are comparable to the commonly used muscle larval ES antigens. The rTsEla is a worthy candidate for early serodiagnosis of trichinellosis and could be a potential alternative antigen to the ML ES antigens. However, more serum samples from patients at an early stage of *Trichinella* infection are needed to further ascertain its sensitivity and specificity.

## Data Availability

The data supporting the conclusions of this article are included within the article.

## Abbreviations

AW: adult worms
BSA: bovine serum albumin
DAB: 3,3′-diaminobenzidine tetrahydrochloride
dpi: days post-infection
ES: excretion/secretion
FBS: fetal bovine serum
ICT: International Commission on Trichinellosis
IFA: immunofluorescence assay
IIL: intestinal infective larvae
ML: muscle larvae
MP: maximum parsimony
MW: molecular weight
NBL: newborn larvae
OD: optical density
OPD: o-phenylenediamine dihydrochloride
TBST: TBS-0.5% Tween 20
TsEla: *T. spiralis* elastase-1
